# Effect of different treatment modalities on the prognosis of patients with stage IIIC cervical cancer

**DOI:** 10.3389/fonc.2024.1405778

**Published:** 2024-05-28

**Authors:** Xiaoman Su, Jiazhen Huang, Ning Wang

**Affiliations:** Department of Gynaecology, The Second Affiliatied Hospital of Dalian Medical University, Dalian, China

**Keywords:** stage IIIC cervical cancer, surgery, radiotherapy, lymph node metastasis, cervical cancer prognosis

## Abstract

**Objective:**

To compare the effects of different treatments on the prognosis of patients with stage IIIC cervical cancer and to identify the main influencing factors to predict the outcomes of patients.

**Methods:**

In this study, a total of 1763 patients with stage IIIC cervical cancer from 2010-2015 were retrospectively analyzed, and these patients were divided into the radical radiotherapy ± chemotherapy group (877 patients) and the radical surgery + radiotherapy ± chemotherapy group (886 patients) according to the treatment methods. The survival differences between the two groups were compared using the Kaplan-Meier method. Unifactorial and multifactorial COX analyses screened the clinical factors affecting the prognosis. The nomogram was constructed, and the accuracy of the line graph was verified using the C-index, calibration, and ROC (receiver operator characteristic curve, ROC).

**Results:**

Age, race, T-stage, pathologic type, mass size, whether or not they underwent surgery, and whether or not they received radiotherapy were independent factors affecting Overall Survival (OS). For all patients with TxN1M0 in cervical cancer stage IIIC, radical synchronized radiotherapy was better than the radical surgery group (p<0.0001). After comparing the tumor size breakdown, it could be found that in the T1N1M0, T2N1M0, and T3N1M0 groups, none of the OS in the surgical group achieved an improvement in OS compared with that in the non-surgical group (p>0.05).

**Conclusion:**

In patients with stage IIIC cervical cancer, OS did not improve in the radical surgery group compared with the radical simultaneous radiotherapy group. And surgery did not benefit patients’ survival regardless of tumor size.

## 1 Introduction

Cervical cancer is one of the most common gynaecological malignant cancers, and its incidence rate ranks ninth among all malignant cancers and fourth among female malignant cancers (1), after breast, colorectal and lung cancers (2). In developing countries, the incidence of cervical cancer is about 60 times higher than that in developed countries due to imperfect screening for cervical cancer. The incidence and mortality rates are still showing varying degrees of increase, with the age of onset tending to be younger, so there is a need to strengthen HPV vaccination, screening for precancerous lesions, and early diagnosis and treatment of cervical cancer (2, 3).

Before 2018, the cervical cancer stage was determined based on clinical examination, supplemented by imaging. As more and more studies have shown, the presence of lymph node metastasis and the number of metastases significantly affects the prognosis of patients. Therefore, the 22nd annual meeting of the International Federation of Gynecology and Obstetrics (FIGO) in October 2018 announced new staging criteria for cervical cancer (4). The new staging system incorporates lymph node metastasis into the diagnostic criteria for stage III cervical cancer, takes into account pelvic and para-abdominal aortic lymph node metastasis, and is subdivided into stages IIIC1 and IIIC2 based on the location of metastasis. The new staging system allows clinicians to clarify lymph node metastasis through imaging or pathology and uses “r” and “p” to indicate imaging or pathology, respectively. If there is a disagreement between the two examinations in determining the current staging, the condition will be attributed to the earlier staging (5). Currently, clinicians’ assessment of lymph node metastasis relies mainly on imaging tests, including ultrasound, computed tomography, magnetic resonance imaging, and positron emission tomography imaging, which play an important role in diagnosing and staging lymph node metastasis (6, 7).

The prognosis of cervical cancer patients with stage III C is significantly heterogeneous, and even with the same staging, their prognosis still has more prominent differences. In this study, we retrospectively analyzed the prognosis of patients with stage III C cervical cancer using a new staging method, identified the key factors affecting prognostic survival, and drew a column-line diagram to predict the outcomes of patients based on their clinical characteristics. This study will provide physicians with valuable decision support and personalized disease management tools for patients.

## 2 Materials and methods

### 2.1 Patient inclusion and exclusion criteria

The patient data for this study were obtained from SEER&Stat version 8.4.1, database Incidence-SEER Research Plus Data, 17 Registries, Nov 2021 Sub (2000-2019). The data of 1763 patients diagnosed with cervical cancer were screened for preliminary analysis from 2010-2015.

Inclusion criteria: 1) Age:18-85 years; 2) Pathologic diagnosis: squamous cell carcinoma, adenocarcinoma, or adenosquamous carcinoma of the uterine cervix; and 3) Clinical stage: stage III (based on the 2018 FIGO staging criteria). Exclusion criteria: 1) Lack of detailed description of the tumors and lymph nodes; 2) Radiotherapy without concurrent chemotherapy; 3) A history of other malignancies.

### 2.2 Statistical analysis

The study was statistically analyzed using SPSS 25.0 and R 4.2.3. Count data were described using frequency counts and analyzed statistically using the χ^2^ test; prognostic factors were screened using univariate and multivariate Cox proportional risk regression models; the Kaplan-Meier method plotted survival curves, and the Log-rank test was used to compare between-group differences. p < 0.05 indicates a statistically significant difference.

## 3 Results

### 3.1 Clinical case information

**Figure 1:**
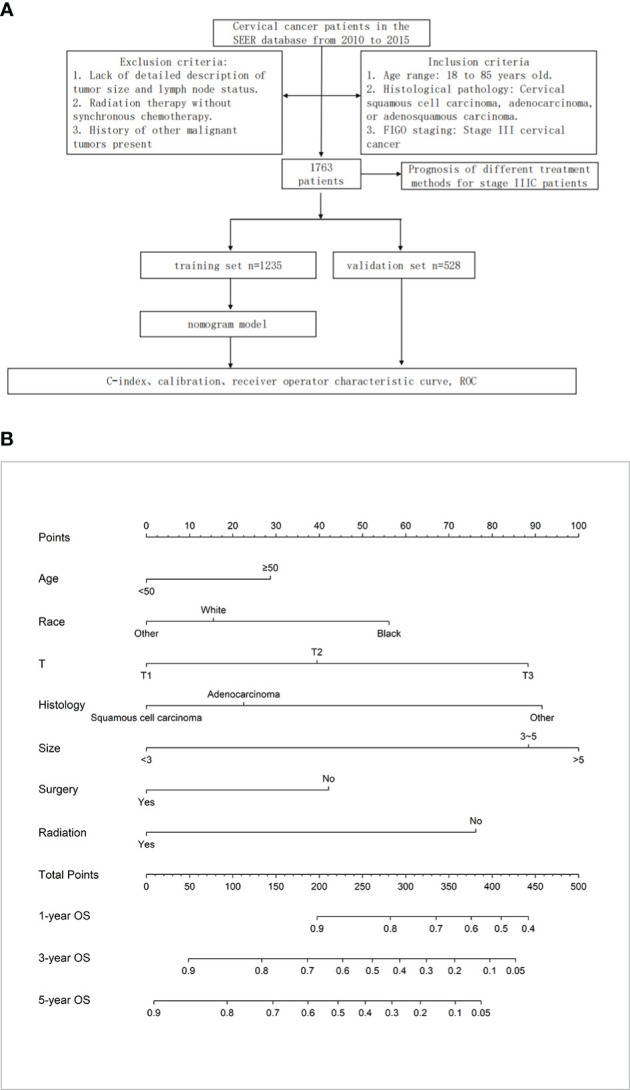
**(A)** Materials and Methods. **(B)** The nomogram for OS in patients with stage IIIC cervical cancer.

A total of 1763 patients were included in this study, and we statistically described them in terms of ethnicity, marital status, stage, differentiation status, pathological type, tumor size, and treatment modality, respectively. Among them, Caucasians accounted for about 75.61%, unmarried patients accounted for approximately 57.35%, patients with T1 stage accounted for approximately 40.61%, patients with moderate to low differentiation accounted for approximately 90.86%, patients with squamous carcinoma accounted for approximately 72.15%, and patients with a mass larger than 5 cm accounted for about 45.72%. In addition, there was little difference among the treatment modalities between surgical and non-surgical patients, with more radiotherapy patients than non-radiotherapy patients (90.47%) and more chemotherapy patients than non-chemotherapy patients (87.52%) (**Figure 1A**).

We randomized these 1763 patients into two groups in a ratio of 7:3, with 1235 patients in the training set and 528 patients in the validation set. There was no significant difference in the clinical data of these two groups regarding baseline balance (P > 0.05), as detailed in **Table 1**.

**Table 1:** Baseline characteristics in two cohorts.

Characteristics	All N = 1763	Training cohort N = 1235	Validation cohort N = 528	p
**Age**				0.916
<50	970 (55.02)	681 (55.14)	289 (54.73)	
≥50	793 (44.98)	554 (44.86)	239 (45.27)	
**Race**				0.311
Black	216 (12.25)	147 (11.90)	69 (13.07)	
White	1333 (75.61)	946 (76.60)	387 (73.30)	
Other	214 (12.14)	142 (11.50)	72 (13.64)	
**Marital**				0.940
Unmarried	1011 (57.35)	707 (57.25)	304 (57.58)	
Married	752 (42.65)	528 (42.75)	224 (42.42)	
**T**				0.537
T1	716 (40.61)	492 (39.84)	224 (42.42)	
T2	659 (37.38)	471 (38.14)	188 (35.61)	
T3	388 (22.01)	272 (22.02)	116 (21.97)	
**Grade**				0.173
I	90 (5.10)	62 (5.02)	28 (5.30)	
II	728 (41.29)	526 (42.59)	202 (38.26)	
III	874 (49.57)	593 (48.02)	281 (53.22)	
IV	71 (4.03)	54 (4.37)	17 (3.22)	
**Histology**				0.785
Adenocarcinoma	304 (17.24)	211 (17.09)	93 (17.61)	
Squamous cell carcinoma	1272 (72.15)	889 (71.98)	383 (72.54)	
Other	187 (10.61)	135 (10.93)	52 (9.85)	
**Size**				0.642
<3	305 (17.30)	216 (17.49)	89 (16.86)	
3~5	652 (36.98)	448 (36.28)	204 (38.64)	
>5	806 (45.72)	571 (46.23)	235 (44.51)	
**Surgery**				0.718
No	848 (48.10)	598 (48.42)	250 (47.35)	
Yes	915 (51.90)	637 (51.58)	278 (52.65)	
**Radiation**				0.748
No	168 (9.53)	120 (9.72)	48 (9.09)	
Yes	1595 (90.47)	1115 (90.28)	480 (90.91)	
**Chemotherapy**				0.140
No	220 (12.48)	164 (13.28)	56 (10.61)	
Yes	1543 (87.52)	1071 (86.72)	472 (89.39)	

### 3.2 Prognostic analysis of cervical cancer patients affected by stage IIIC

As shown in **Table 2**, different clinical indicators were subjected to univariate Cox analysis in the training group. The results showed that age, race, marital status, T stage, pathological type, tumor diameter, degree of differentiation, whether to operate or not, and whether to radiate or not were prognostic correlates of patients with stage IIIC cervical cancer (P < 0.05); the above predictive correlates were further included in multifactorial Cox analysis, and the results showed that Age, race, T stage, pathologic type, mass size, whether surgery, and whether radiotherapy were independent risk factors for patients with stage IIIC cervical cancer (P < 0.05). The results of multifactorial Cox analysis showed that patients older than 50 years old had a poorer prognosis than those younger than 50 years old (HR=1.288, 95% CI: 1.081 to 1.534, P=0.005); Caucasian patients had a relatively better prognosis relative to blacks (HR=0.729, 95% CI: 0.575 to 0.925, P=0.009); and surgical patients had a somewhat better prognosis close to non-surgical patients (HR=0.698, 95% CI: 0.567 to 0.858, P=0.001), radiotherapy patients had a relatively better prognosis relative to non-radiotherapy patients (HR=0.506, 95% CI: 0.386 to 0.664, P<0.001), and T2 and T3 patients had a poorer prognosis close to T1 patients, and the diameter of the mass The larger patients had a relatively poorer prognosis.

**Table 2:** Prognostic analysis of cervical cancer patients affected by stage IIIC.

	Univariate			Multivariate		
	HR	95%CI	p	HR	95%CI	p
Age						
<50	reference					
≥50	1.508	1.273-1.786	<0.001	1.288	1.081-1.534	0.005
Race						
Black	reference					
White	0.594	0.471-0.750	<0.001	0.729	0.575-0.925	0.009
Other	0.534	0.380-0.750	<0.001	0.660	0.466-0.936	0.020
Marital						
Unmarried	reference					
Married	0.838	0.705-0.995	0.044	0.861	0.721-1.027	0.097
T						
T1	reference					
T2	1.842	1.492-2.275	<0.001	1.435	1.142-1.802	0.002
T3	3.215	2.583-4.003	<0.001	2.240	1.749-2.869	<0.001
grade						
G1	reference					
G2	0.754	0.510-1.114	0.156	0.798	0.536-1.188	0.266
G3	1.080	0.737-1.583	0.692	0.995	0.675-1.468	0.982
G4	1.755	1.076-2.863	0.024	1.567	0.95-2.582	0.078
Histology						
Adenocarcinoma	reference					
Squamous cell carcinoma	0.956	0.764-1.197	0.697	0.853	0.674-1.079	0.184
Other	1.653	1.226-2.227	0.001	1.704	1.257-2.311	0.001
Size						
<3	reference					
3~5	2.812	2.008-3.937	<0.001	2.151	1.516-3.051	<0.001
>5	3.911	2.820-5.425	<0.001	2.365	1.644-3.402	<0.001
Surgery						
No	reference					
Yes	0.508	0.427-0.603	<0.001	0.698	0.567-0.858	0.001
Radiation						
No	reference					
Yes	0.627	0.487-0.807	<0.001	0.506	0.386-0.664	<0.001
Chemotherapy						
No	reference					
Yes	0.793	0.625-1.007	0.057			

### 3.3 Construction and verification of the nomogram for cervical cancer

**Figure 2:**
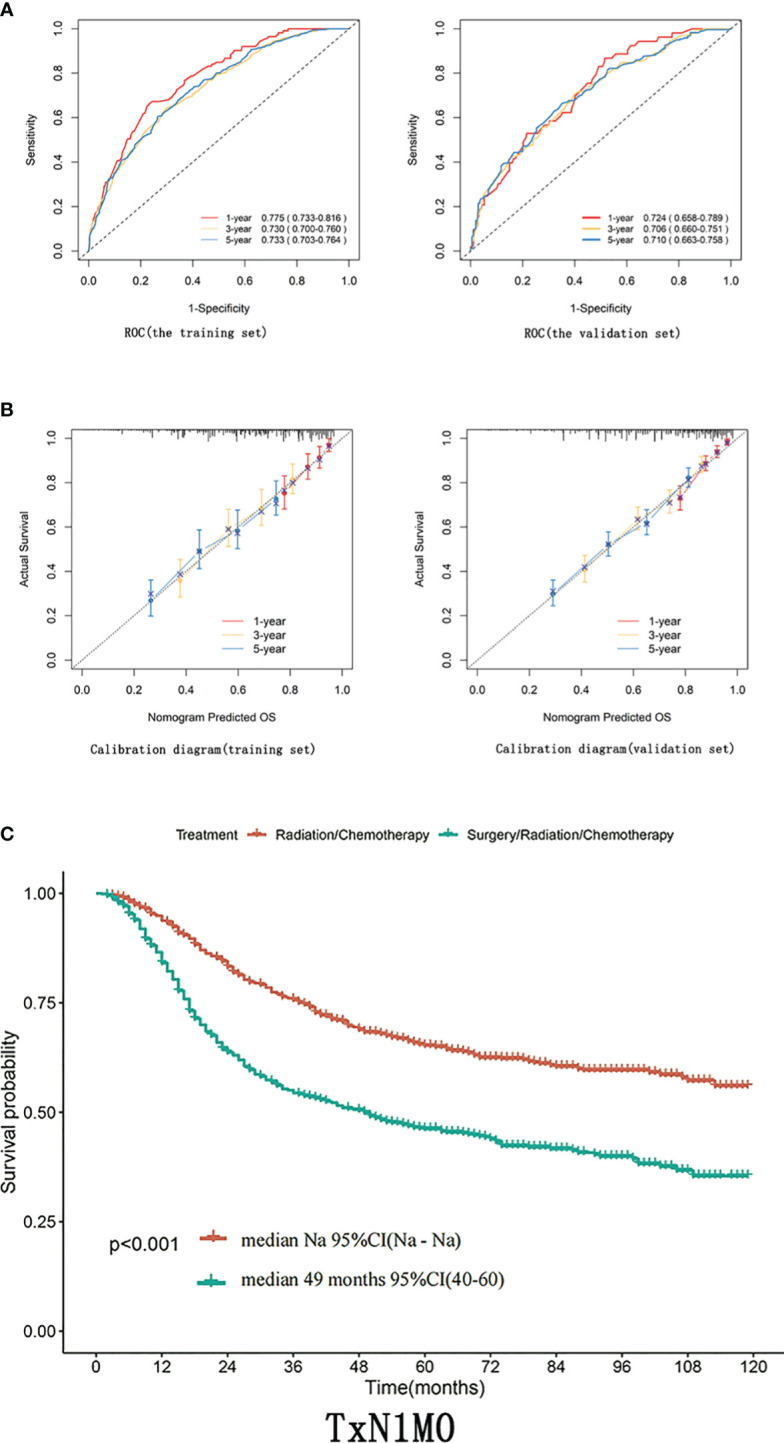
**(A)** ROC curves of 1-year, 3-year, 5-year, and 8-year OS for training and validation sets. **(B)** Calibration curves for 1-year, 3-year, and 5-year OS of training and validation sets. **(C)** In patients with TxN1M0 (**Figure 2C**), radical synchronous radiotherapy was better than the radical surgery group.

Based on multifactorial analysis, age, race, T stage, mass size, histologic type, surgery, and radiotherapy were included in the model, and a nomogram for OS in patients with stage IIIC cervical cancer was successfully constructed. The tumor size contributed the most to the column-line graph, followed by histological type, T-stage, radiotherapy, race, surgery, and age (**Figure 1B**). The C-index was 0.687 (0.665-0.709) in the training set and 0.666 (0.635-0.698) in the validation set. The AUCs of 1, 3, and 5 years were respectively 0.775, 0.730, and 0.733 in the training set, and the AUCs of 1, 3, and 5 years were 0.724, 0.706, and 0.710 in the validation set (**Figures 2A, B**).

### 3.4 Evaluating the prognosis of stage IIIC patients with different treatment modalities based on T-staging

**Figure 3:**
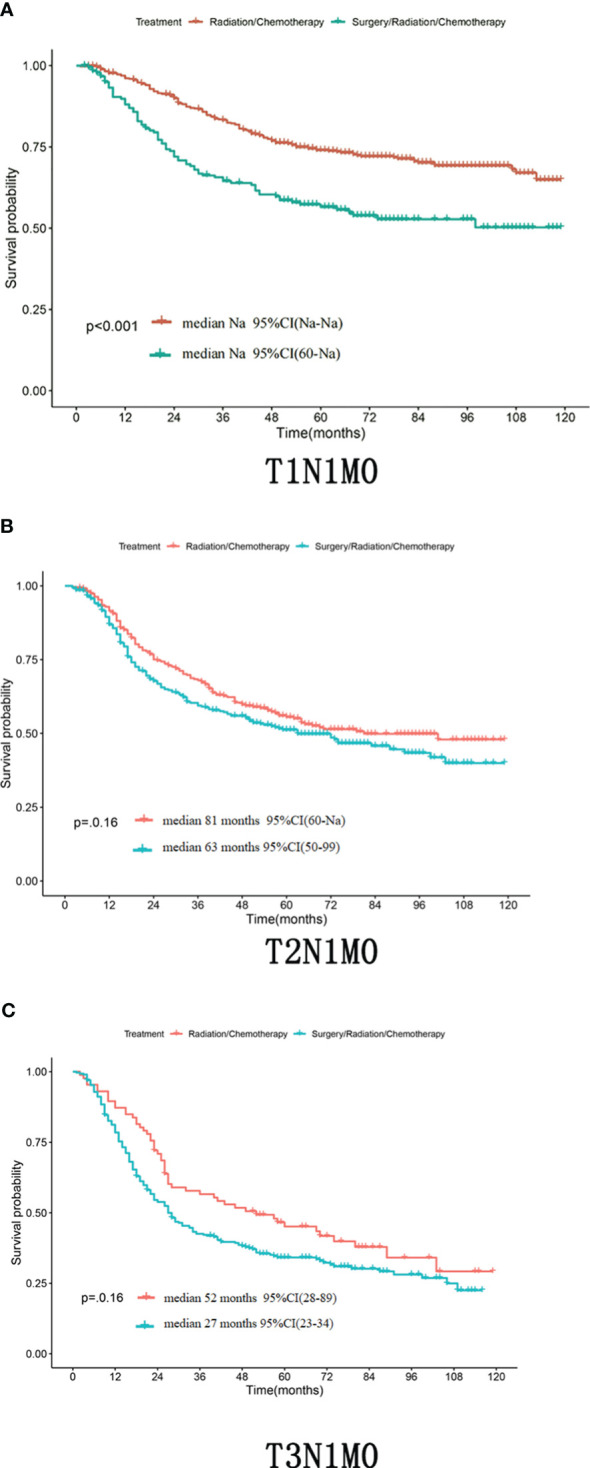
**(A–C)** Whether in the patient with T1N1M0,T2N1M0 or T3N1M0,the OS of the radical synchronous radiotherapy group was higher than that of the radical surgery group.

Differences in T staging are important factors affecting the prognosis of stage IIIC patients. In patients with TxN1M0 (**Figure 2C**), radical synchronous radiotherapy was better than the radical surgery group. Still, such a comparison had a large bias, so we performed a subdivided comparison, and the radical synchronous radiotherapy group was higher than that of the radical surgery group.Regardless in patients with stage T1 (T1N1M0),T2 (T2N1M0) or T3 (T3N1M0),extreme cervical cancer (C-type hysterectomy) and pelvic lymph node dissection ± para-abdominal aortic When comparing the OS of radical lymph node dissection + postoperative adjuvant synchronous radiotherapy with radical synchronous radiotherapy, the radical synchronous radiotherapy group was higher than that of the radical surgery group (**Figures 3A–C**). For patients with stage IIIC cervical cancer, in the TxN1M0 group, OS did not improve in the radical surgery group compared with that in the radical synchronous radiotherapy group, and surgery did not provide additional benefit to patients.

## 4 Discussion

The purpose of continuously improving cervical cancer staging is to standardize clinical treatment. Staging assesses the severity and prognosis of cervical cancer based on factors such as tumor size, lymph node metastasis, and distant metastasis. The latest 2018 International Federation of Gynecology and Obstetrics (FIGO) staging of cervical cancer incorporates stage IIIC into the staging to address the prognostic impact of lymph node metastasis. The staging system before 2018 did not explicitly include stage IIIC, which led to disagreements in the treatment choices and predictive assessments for these patients. The new staging system, however, allows for more accurate treatment and prognostic evaluation of patients with stage IIIC cervical cancer; for patients with stage IIIC cervical cancer, radical radiotherapy plus concurrent platinum-containing chemotherapy has been shown to improve survival (8, 9). Radical radiotherapy destroys tumor cells using radiation exposure, while platinum-containing chemotherapy is efficacious by inhibiting the division and proliferation of cancer cells. Several studies have demonstrated the effectiveness of radical radiotherapy plus concurrent platinum-containing chemotherapy, and it has become the standard of care for patients with stage IIIC cervical cancer. For example, the National Comprehensive Cancer Network (NCCN) guidelines have identified radical radiotherapy plus concurrent platinum-based chemotherapy as the standard of care for stage III cervical cancer (10).

According to FIGO’s new staging system, the treatment recommendation for stage III C is radical synchronized radiotherapy, and stratified treatment is not recommended. However, some experts recommend surgery + radiotherapy for patients with stage III C1 (11). Concerning the necessity of radical surgery, several studies have now concluded that surgery is not necessary (12–15), and they have noted that radical surgery may not be necessary for some patients with stage III C. Several studies have shown that surgical treatment did not significantly prolong survival or improve prognosis in patients receiving concurrent radiotherapy. In addition, surgery may result in more surgery-related complications and decreased quality of life. Therefore, they suggested that when deciding whether to undergo surgery, patients’ conditions should be assessed individually, the potential benefits and risks of surgery should be considered, and the most appropriate treatment option should be selected. There is disagreement about the specific treatment options for some stage IIIC. It has been found that patients face a higher risk when they opt out of radical surgery. It may be difficult to control the patient’s localized lesions effectively, leading to decreased recurrence rates and disease-free survival. In addition, patients who forgo surgery are more likely to experience severe radiotherapy side effects compared to those who complete radical surgery, which may be related to earlier initiation of concurrent radiotherapy (16, 17). In a retrospective multicenter study conducted across multiple regions, researchers analyzed 515 patients who were found to have positive lymph nodes after undergoing radical surgery for cervical cancer. The results showed that radical surgery with complete hysterectomy did not show a benefit in terms of improved survival for patients with cervical cancer in the presence of lymph node metastases, regardless of tumor size or histologic type. In contrast, opting out of radical surgery not only avoids the double treatment burden of surgery plus radiotherapy but also significantly reduces surgical risks and complications. Therefore, in the case of intraoperative confirmation of the presence of lymph node metastases, it is recommended to forgo radical surgery and instead suggest a treatment regimen of radical simultaneous radiochemotherapy (18). Another retrospective study analyzed the results, showing that resectioning patients’ pelvic lymph nodes and enlarged lymph nodes adjacent to the abdominal aorta significantly improved survival rates (19). The number of lymph nodes and the degree of spread reflect the degree of malignancy and invasiveness of the tumor, and studies have shown that regardless of whether the patient receives surgical treatment or radical simultaneous radiotherapy, showing that the survival rate decreases progressively as the number of lymph node metastases increases (20, 21). For patients with stage IIIC cervical cancer, this study aimed to compare the prognostic impact of different treatment modalities and to utilize a nomogram to develop a predictive model to assess the probability of patient survival. In recent years, the method of building cancer prediction models based on a nomogram has been widely used to predict the likelihood of a specific event. It can effectively predict survival differences between different individuals. By using the nomogram, the prognosis of patients with cervical cancer can be better assessed, and appropriate regimens can be developed based on individual patient characteristics and treatment modalities. The results of this study are important for guiding clinical decision-making and improving the outcome of cervical cancer patients (22). The nomogram established in this study was experimentally demonstrated to have high predictive ability. In addition, in assessing the model’s accuracy, we used the method of calibration curves. In this study, the results of the calibration curves will provide an important reference for us to validate the reliability and application prospects of the column-line diagram model further (23). Further subgroup analyses revealed that for patients with stage IIIC cervical cancer, as seen in the TN1M0 group, no improvement in OS was achieved in the radical surgery group compared with the radical simultaneous radiotherapy group, and surgery did not result in additional benefit to patients. Current clinical practice differs in the management of patients with intraoperative lymph node involvement; according to European guidelines, it is recommended that further pelvic surgery should be avoided if lymph node involvement is detected intraoperatively, but this view is not supported by strong evidence, and only case studies have been reported. This study showed that completing a radical hysterectomy did not improve the prognosis of patients with intraoperatively detected lymph node involvement; furthermore, the risk of recurrence was not reduced, regardless of tumor size, histologic type, or other traditional risk factors. Therefore, patients who have pelvic lymph node involvement detected intraoperatively should be considered to forgo radical surgical treatment and opt for radical simultaneous radiotherapy (18).

This study is a retrospective analysis with some limitations. In the study, we randomly included eligible cases for analysis, which aligns with the clinical reality. However, there was an imbalance in the staging of the sample, with most of the cases being stage IIIC patients. In addition, the two treatment groups had a large difference in numbers. These factors may have biased the results to a certain extent. Therefore, to assess the predictive effects of different treatment modalities accurately further, studies using larger sample sizes are needed to validate the findings. In addition, multicenter studies are required to extend our findings and explore the factors influencing treatment effects more comprehensively. Moreover, due to the lack of detailed information on surgery and radiotherapy in the database such as Querleu-Morrow classification of surgery, laparoscopic or open surgery, and the specific dosage of radical radiotherapy,which may have an impact on the prognosis of cervical cancer patients,further stratified analysis based on this information was not conducted (24–26). So further researches are required.These further studies will help to improve our predictive assessment and choice of treatment options for patients with cervical cancer.

In summary, we successfully constructed a prognostic overall survival column chart based on several prognostic factors of cervical cancer, such as gender, race, T-stage, pathologic type, mass size, whether surgery was performed or not, whether radiotherapy was performed or not, etc., which were obtained from the results of multifactorial analysis. This column chart can help gynaecologists more accurately assess the prognosis of patients and provide guidance for clinical individualized treatment. By analyzing the effects of multiple factors, we were able to understand the predictive risk of cervical cancer patients more comprehensively. We transformed it into a visual column chart, enabling physicians to understand patients’ prognosis more intuitively. However, to further validate the predictive ability and accuracy of the column-line diagram, we need to validate it in a larger sample size and multi-central study to improve the reliability and wide applicability of the model. When considering the treatment options for patients with stage IIIC cervical cancer, we need to consider the risks and economic burdens associated with surgery comprehensively. Although radical surgery can effectively control localized lesions, for patients with stage IIIC cervical cancer, the scope of surgical resection may be more extensive, requiring removal of the uterus, adnexa, and pelvic lymph nodes, which will increase the complexity and risk of surgery. In addition, some patients may be at risk for postoperative complications, such as bleeding, infection and urinary incontinence. In addition, surgery requires a longer recovery time and investment of medical resources, which can be financially burdensome for patients.

In contrast, radical radiotherapy is a more conservative and integrative treatment option. It can effectively control locally advanced cervical cancer with lower surgical risk and recovery time. Radiotherapy is a combination of external radiation therapy and chemotherapy, which can prevent tumor cells as well as inhibit their recurrence and metastasis through radiation therapy. Chemotherapeutic agents can eliminate tumor cells through targeted therapy and cytotoxicity. Radical radiotherapy is expected to control localized lesions and reduce the risk of recurrence. Although radical radiotherapy may cause some adverse effects, such as nausea, vomiting, malaise, and bone marrow suppression, these effects are usually manageable and temporary. They can be managed with appropriate supportive therapy. In addition, radical radiotherapy can reduce the financial burden associated with surgery. Although radical radiotherapy is a more recommended treatment option, we must still validate its efficacy and safety in further clinical studies and evaluate its long-term effects on patients. At the same time, patient individualization factors and preferences should also be fully considered to develop the most appropriate treatment plan for patients.

## Data availability statement

The datasets presented in this study can be found in online repositories. The names of the repository/repositories and accession number(s) can be found in the article/supplementary material.

## Ethics statement

Ethical approval was not required for the study involving humans in accordance with the local legislation and institutional requirements. Written informed consent to participate in this study was not required from the participants or the participants’ legal guardians/next of kin in accordance with the national legislation and the institutional requirements.

## Author contributions

XS: Writing – review & editing, Writing – original draft. JH: Writing – review & editing. NW: Writing – review & editing, Supervision, Project administration.
